# Size-conditional smolting and the response of Carmel River steelhead to two decades of conservation efforts

**DOI:** 10.1371/journal.pone.0188971

**Published:** 2017-11-30

**Authors:** Juan Lopez Arriaza, David A. Boughton, Kevan Urquhart, Marc Mangel

**Affiliations:** 1 Department of Applied Mathematics and Statistics, University of California, Santa Cruz, California, United States of America; 2 Center for Stock Assessment Research, University of California, Santa Cruz, California, United States of America; 3 Southwest Fisheries Science Center, National Marine Fisheries Service, National Oceanic and Atmospheric Administration, Santa Cruz, California, United States of America; 4 Monterey Peninsula Water Management District, Monterey, California, United States of America; 5 Department of Biology, University of Bergen, Bergen, Norway; Department of Agriculture and Water Resources, AUSTRALIA

## Abstract

Threshold effects are common in ecosystems and can generate counterintuitive outcomes in management interventions. A threshold effect proposed for steelhead trout (*Oncorhynchus mykiss*) is size-conditional smolting and marine survival. Steelhead are anadromous, maturing in the ocean but migrating to freshwater to spawn, where their offspring reside for one or more years before smolting—physiologically transforming to a saltwater form—and migrating to the ocean. In conditional smolting, juveniles transform only if growth exceeds a threshold body size prior to migration season, and subsequent marine survival correlates with size at ocean entry. Conditional smolting suggests that efforts to improve freshwater survival of juveniles may reduce smolt success if they increase competition and reduce growth. Using model-selection techniques, we asked if this effect explained declining numbers of adult Carmel River steelhead. This threatened population has been the focus of two decades of habitat restoration, as well as active translocation and captive-rearing of juveniles stranded in seasonally dewatered channels. In the top-ranked model selected by information-theoretic criteria, adult decline was linked to reduced juvenile growth rates in the lower river, consistent with the conditional smolting hypothesis. According to model inference, since 2005 most returning adult steelhead were captively-reared. However, a lower-ranked model without conditional smolting also had modest support, and suggested a negative effect of captive rearing. Translocations of juvenile fish to perennial reaches may have reduced the steelhead run slightly by raising competition, but this effect is confounded in the data with effects of river flow on growth. Efforts to recover Carmel River steelhead will probably be more successful if they focus on conditions promoting rapid growth in the river. Our analysis clearly favored a role for size-conditional smolting and marine survival in the decline of the population, but did not definitively rule out alternative explanations.

## Introduction

Recovery of endangered Pacific salmon and steelhead in the USA is a far-reaching effort, with the goal of reversing a century-long decline of wild salmonid fish populations [1, 2] throughout a large coastal region [3–8]. A key theme in the original decline of salmonids was a lack of suitable analysis asking if scientifically-based management strategies were working as anticipated. Critics argue that dysfunctional patterns of political and economic decision-making tended to favor scientists who over-stated their understanding and under-analysed the response of salmon stocks to management interventions [1, 2], and that this desire for simple answers helped enable a societal process driving the decline [1]. In general, ecological systems feature non-linear dynamics and threshold effects whose response to management cannot be fully predicted [9], especially when coupled to social systems [10]. Pacific salmonids are no exception [11–13], and to prevent the mistakes of the past, their recovery requires an iterative process of learning from mistakes and altering recovery efforts accordingly. Thus, a fundamental scientific need is retrospective analysis of how populations respond to conservation actions—often multiple actions taken concurrently.

Threshold effects are defined as small changes in an environmental driver that produce a large response in an ecosystem, often with effects that are large and profound, but difficult to predict or manage [14]. Two potentially important threshold effects in populations of steelhead (*Oncorynchus mykiss*) are size-conditional smolting and size-conditional survival during the early marine phase of the species’ lifecycle [15]. Smolting is the process in which juvenile salmonids in freshwater physiologically transform into saltwater-tolerant fish that migrate downstream to the ocean, where they grow and mature into adults, eventually returning to freshwater to spawn. This life-history pattern is known as anadromy. In size-conditional smolting, the transformation is conditional on realized growth and lipid storage exceeding internal physiological thresholds during a seasonal “decision window” (thought to occur in the fall or winter prior to the spring migration season [16–18]). Realized growth and lipid storage represent an integration of various environmental factors, such as temperature, food availability, competition, and activity patterns. In contrast, the physiological size threshold is thought to be a product of natural selection, with genetically-determined thresholds varying from region to region as a function of local adaptation [19, 20].

Once smolts enter the ocean, their survival in the early marine phase is typically correlated with size at ocean entry [21, 22]. Using scale analysis, Ward and Slaney [22] found that marine survival of smolts from the Keogh River in British Columbia increased steadily from ~3% for smolts of 140 mm length up to an apparent asymptote of ~40% survival at lengths > 250 mm. Similarly, in a coastal California creek, Bond et al. [21] found that very few smolts shorter than 150 mm returned as adults despite the vast majority of downstream migrants having shorter lengths, and suggested that 150 mm was a threshold for survival. Although correlated size and survival do not automatically imply threshold effects, the range 140 mm–250 mm, where improvement in survival is observed, comprises about 15% of the total length increase from fry to adulthood. This suggests a sharp increase in survival, and hence production of anadromous adults, within a relatively narrow window of realized growth, consistent with the definition of threshold effect. Similar effects are observed in Atlantic salmon, *Salmo salar* [18]. The mechanism for the sharp increase in survival with size is not clear, but increased size confers many advantages on salmonids, including swim speed, which scales with body length, and may involve threshold burst speeds beyond which fish can exploit new prey species or evade important predators [22, 23]. Larger size also helps salmonids resist starvation [24], disease [25], and possibly parasitism [26].

Non-smolting steelhead continue to feed in freshwater, and may be cued to smolt in next year’s decision window, or be cued to mature into reproductive, freshwater-resident adults at an earlier decision window (thought to occur in the spring [17]). Population models based on these concepts have successfully explained variation in life-history patterns of threatened steelhead populations [19, 27]. In principal, even modest changes in the environmental factors that influence growth could shift a population from primarily anadromous (most fish have a marine phase) to primarily freshwater-resident, or vice-versa [28]. This could produce threshold effects at the population level and undesirable management outcomes, for example if efforts to improve the survival of juveniles increased competition for food and thereby reduced growth and thereby the smolting rate or early marine survival, or if restoration of riparian vegetation generated cooler stream temperatures that affected growth rates.

Here, we describe a retrospective analysis of the combined effect of size-conditional smolting and size-conditional marine survival on the abundance of anadromous steelhead in a population subject to two decades of recovery efforts. Carmel River historically supported the largest anadromous steelhead run in the chaparral ecosystems of central and southern California, but development of water and land resources led to habitat degradation and an estimated 75% population decline by 1975 [29]. Depletion of the water table killed extensive stands of riparian vegetation in a drought in the late 1970s [30], triggering a sustained interest in rehabilitating the river and its steelhead. Since then, the river has been a focus of steelhead recovery efforts, including reestablishment of riparian vegetation, improved groundwater management, rehabilitation of the estuary in 2004, removal of a large mainstem dam in 2015, and ongoing plans to restore floodplains, repatriate water withdrawals back to river flows, and possibly remove a second dam. Groundwater pumping still routinely dewaters the lower river in summer, but has been mitigated for 20 years by a combination of captive rearing and within-river translocations of juvenile steelhead rescued from the dewatered sections each summer.

**Fig 1:**
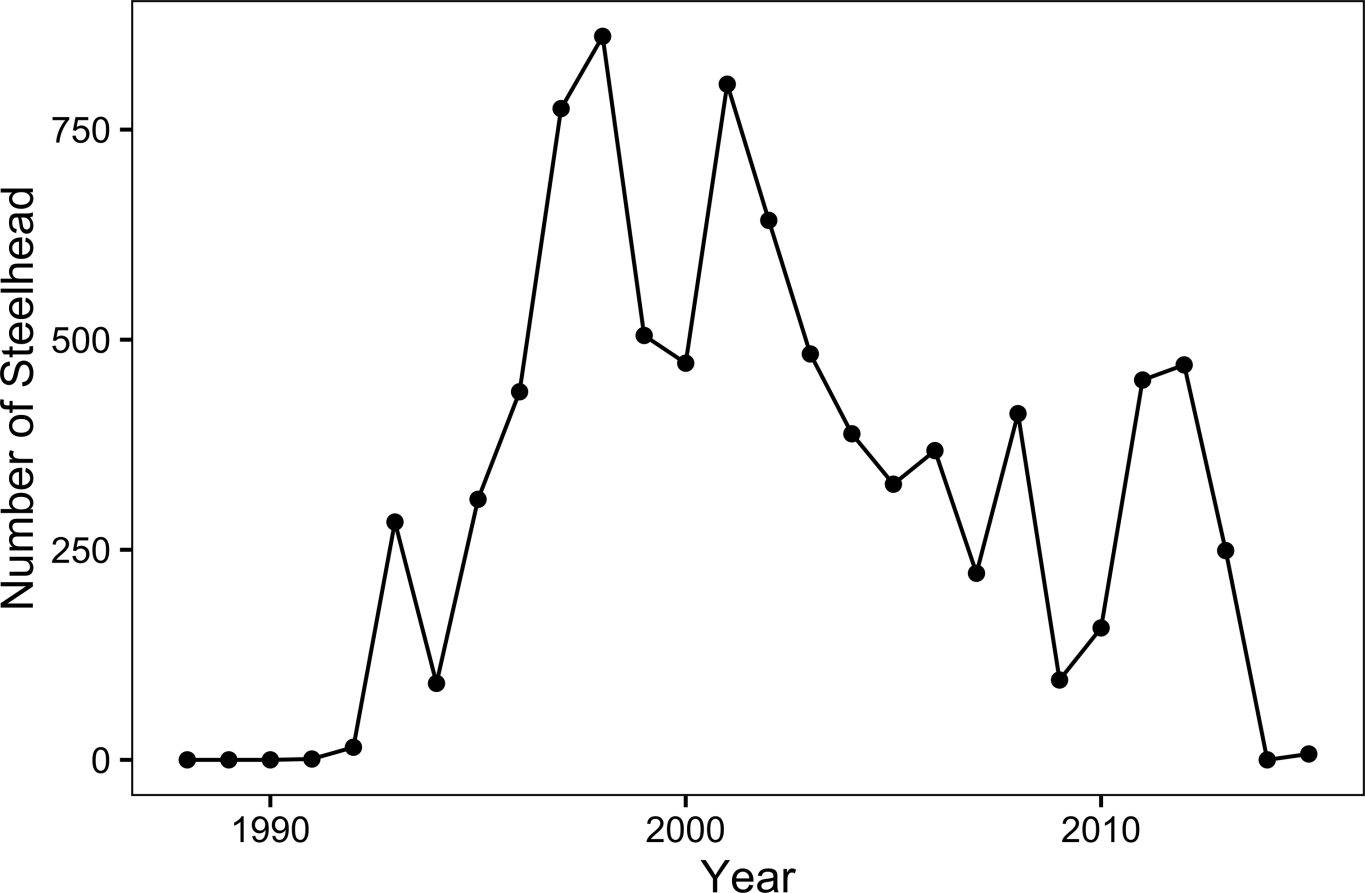
Annual counts of adult steelhead migrating past San Clemente Dam. Droughts combined with water pumping completely prevented migratory access between river and ocean during 1988–1991 and 2014. A captive-broodstock effort by the Carmel River Steelhead Association contributed to the rapid recovery of the run after the 1987–1992 drought. Location of San Clemente Dam is shown in Fig 2.

After the 1987–1992 California drought, the anadromous steelhead abundance initially responded positively to these and other restoration efforts, but since 2001 the number of anadromous adults has shown a variable yet consistent decline (Fig 1). Because sustained conservation efforts failed to generate a long-term increase in the abundance of anadromous adults, we conducted a retrospective analysis focused on how the population has responded to its history of conservation actions. Because of the potential importance of size-conditional smolting and early marine survival, we focused our attention on how these threshold phenomena may interact with efforts to conserve the population by improving juvenile survival and abundance. Our goal here was to evaluate the relative support for different hypotheses for the decline. We address three sets of questions:

1. Is a size-conditional model of smolting and marine survival a better predictor of the abundance of anadromous adults, relative to a fixed-rate model, in which all juveniles have the same probability of smolting and surviving to adulthood?
2. What can we infer about trends in production of anadromous adults from wild-reared versus captively-reared juveniles, and are they more related to changes in juvenile abundance or to rates of size-conditional smolting?
3. Do within-river translocations show a positive relationship with subsequent juvenile abundance, as intended by resource managers? Or do they show a negative relationship with subsequent juvenile abundance or rates of conditional smolting, which is *not* intended but a plausible outcome due to competitive effects? Or is there neither relationship, suggesting translocations have little effect on abundance of anadromous adults?

## Materials and methods

### Study system

**Fig 2:**
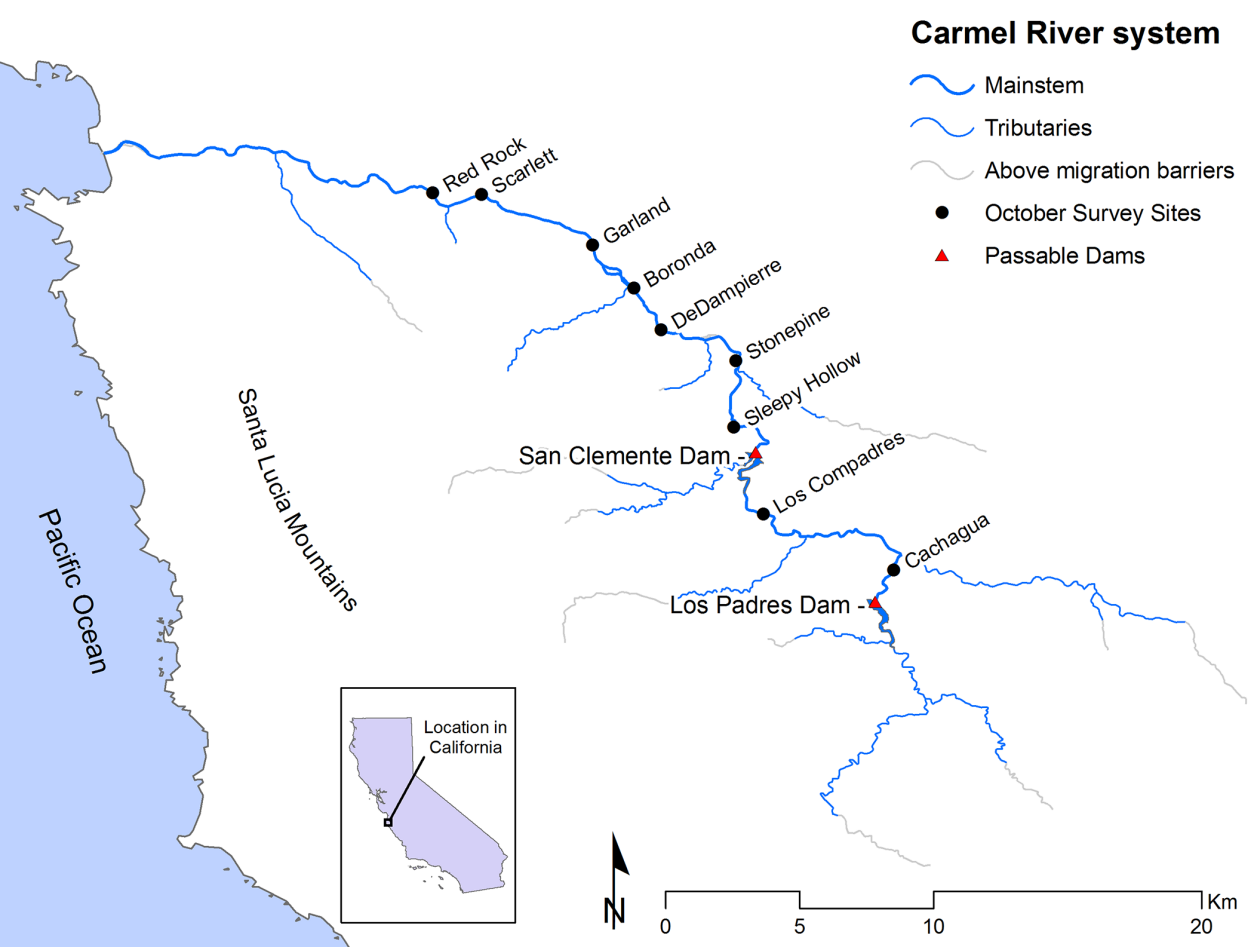
The Carmel River system showing passable dams and sites of juvenile sampling. The Monterey Peninsula Water Management District estimated juvenile abundance and size distributions at the 9 sites from 1996 to present. Counts of migratory adults returning from the ocean to spawn were made at a fish ladder on San Clemente Dam. In summer, large sections of the river between the Garland site and the ocean were routinely dewatered by groundwater extraction; juvenile fish rescued from these sections were either transferred to an off-channel rearing facility (“captively reared”) or simply moved to another part of the river with perennial surface flows (“translocated”).

Carmel River drains a mountainous, 660 km^2^ coastal watershed in California (Fig 2), where the nearby ocean generates a "Mediterranean" climate of warm wet winters and foggy, rainless summers. Adult steelhead typically migrate up from the ocean to spawn January through April; offspring typically remain in the river 1 year (occasionally 2 and rarely 3), then smolt and migrate down to the ocean in April or May the following year [29, 31]. They mostly mature in the ocean for 1 or 2 years and then migrate back to the river to spawn [31]. During our study period, two large dams on the river (Fig 2) provided somewhat-impeded passage for migrating adults, though the lower one (San Clemente Dam) has since been removed. Small numbers of fish mature in the ocean for 3 or 4 years, and a modest proportion of spawners survive to spawn in a subsequent year (iteroparity) [31]. These rarer life-history variants are likely important for population resilience. However, we omitted them from our analysis for two reasons: 1) Our focus was on the major pathways of production, rather than resilience, so we focused on the two most common life-histories (1 year in freshwater and 1 or 2 years in the ocean); and 2) we wanted to keep our model simple, to avoid overfitting by keeping the number of estimated parameters much smaller than the number of datapoints (n = 18 years). The effect of this simplification is that the rarer life-histories are represented as residual variation in our models.

The freshwater-resident form of *O*. *mykiss* (rainbow trout) also occurs in this system, but is rare [31]. Residents are observed mostly in the high-rainfall portion of the watershed upstream of the upper dam, where migration of anadromous adults is impeded by sporadic droughts that, in combination with municipal water usage, dewater the lower river, and by passage difficulties at the upper dam [31]. Dettman and Kelley [31] observed that frequency of residents increased in the watershed above the dam during the drought of 1976–77, but their restricted distribution suggests that most juvenile production in normal years comes from anadromous adults. Recent genetic analyses corroborated this view, finding that 95.8% of juveniles sampled between the two dams had a genetic signature for anadromy [20].

After the completion of a captive-broodstock and reintroduction program [32] during the 1987–1992 California drought (Fig 1), a comprehensive effort of habitat restoration, fish rescues, and population monitoring was initiated. Habitat restoration included re-establishment and management of riparian vegetation killed by the combination of drought and drawdown of the water table. Other accomplishments were restoration of summer surface flows via reduced drawdown of the water table in some reaches, and rehabilitation of portions of the estuary. Since 1989, juvenile steelhead have been rescued annually during the low-flow season (May–Oct) from drying reaches in the mainstem and tributaries, and immediately translocated to other sections of the river with perennial surface flow. Since 1996, a portion of the rescued fish were captively-reared in a specialized facility and released back to the river the following winter, with the remainder being translocated as before. To minimize effects of captively-reared juveniles on wild fish in the river, the captives are released in the lower river near the estuary, generally after winter rainfall has re-established surface flows connecting the river to the ocean for at least two weeks.

### Datasets

All archived data used for this analysis were from activities permitted by the California Department of Fish and Wildlife and the National Marine Fisheries Service. Time-series data available for Carmel River steelhead include the adult counts (Fig 1) and three juvenile datasets describing wild fish, captively-reared fish, and translocated fish, respectively (Table 1).

**Table 1:** Time-Series Data for Carmel River Steelhead, 1996–2013.

Dataset	Description
Adult Abundance	Counts of migrating adults ascending a fish ladder on San Clemente Dam, 29.8 km upstream of the ocean.
Captively-Reared Juveniles	Counts and sizes of captively-reared juveniles, rescued from drying reaches each summer, and released back to the river each winter after full surface-flows are established.
Translocated Juveniles	Counts of translocated juveniles, rescued from drying reaches each summer and immediately translocated to reaches retaining surface flow.
October Juvenile Survey	9 sites between the ocean and Los Padres Dam (40 km upstream of the ocean), surveyed each October via 3-pass depletion-electrofishing. Fish are a mixture of wild and translocated juveniles

Neither the adult abundances nor the October juvenile surveys are unbiased estimates for the entire population. The adult counts omit steelhead spawning downstream of San Clemente Dam, which includes the lower mainstem and five tributaries that together comprise 44.8% of the system’s 111 km of accessible stream habitat. If spawning densities (per stream km) are spatially even throughout the system, then proportional bias of the San Clemente counts should be -0.448.

Some rough support for the “spatially-even” assumption comes from additional counts made at the second dam (Los Padres), and also from two spawning surveys in the mainstem below San Clemente Dam. For the former, the ratio of accessible stream km above Los Padres versus between San Clemente and Los Padres is 0.50, and the corresponding mean ratio for 22 years of steelhead counts is 0.428 (SE = 0.048, *n* = 22 yearly ratios), which is not significantly different from 0.50 (*p* = 0.16 by one-sample t-test; normality of data not rejected by Shapiro-Wilk test, *p* = 0.48). For the spawning surveys (conducted 2007 and 2008), Williams et al. [33] estimated that between 0.2 and 0.6 of spawning occurred in the mainstem below San Clemente Dam (omitting tributaries below the dam), whereas the corresponding ratio of stream km is within this range, at 0.326.

The October survey sites are biased because they omit tributaries and the section of mainstem above Los Padres Dam. These unsurveyed reaches comprise 61.5% of total stream km, although we expect the proportional bias would be weaker (> -0.615) since tributary channels are generally narrower than the mainstem and thus probably have lower numbers of fish per unit length.

In our model-development, we estimate “apparent” transition rates, defined as estimates of the transition rates (here, from the juvenile stage to the adult stage) that incorporate these biases. However, in general the apparent transition rates confound bias with estimates of vital rates (stage-specific survival, smolting rates, and fecundity), and need to be interpreted accordingly (see smolting model section below). The key assumption of this approach is that the bias in a given time-series varies little through time, so that among-year variation in counts is attributed to variation in vital rates, and model selection thus reflects underlying population processes.

### Analytical approach

In general, we addressed our questions by developing contrasting models, fitting them to the data, and selecting a best approximating model using AICc (Akaike’s Information Criterion, corrected for small samples [34, 35]). The model with lowest AICc score was selected as most likely to be the best-approximating model.

Following Burnham and Anderson [34], differences in AICc between the most-likely model and other models (Δ_*i*_) were used to judge relative support: Δ_*i*_ < 2 was interpreted as substantial support for model *i* despite it not having the lowest score, whereas Δ_*i*_ > 4 indicated considerably less support than the most-likely model and Δ_*i*_ > 10 indicated essentially no support [34]. In addition, we calculated model weights and evidence ratios, respectively interpreted as the probability that model *i* was the best-approximating model, and as the odds against model *i* relative to the most-likely model [34].

### Estimating ages, sizes and abundance of juveniles in October

**Fig 3:**
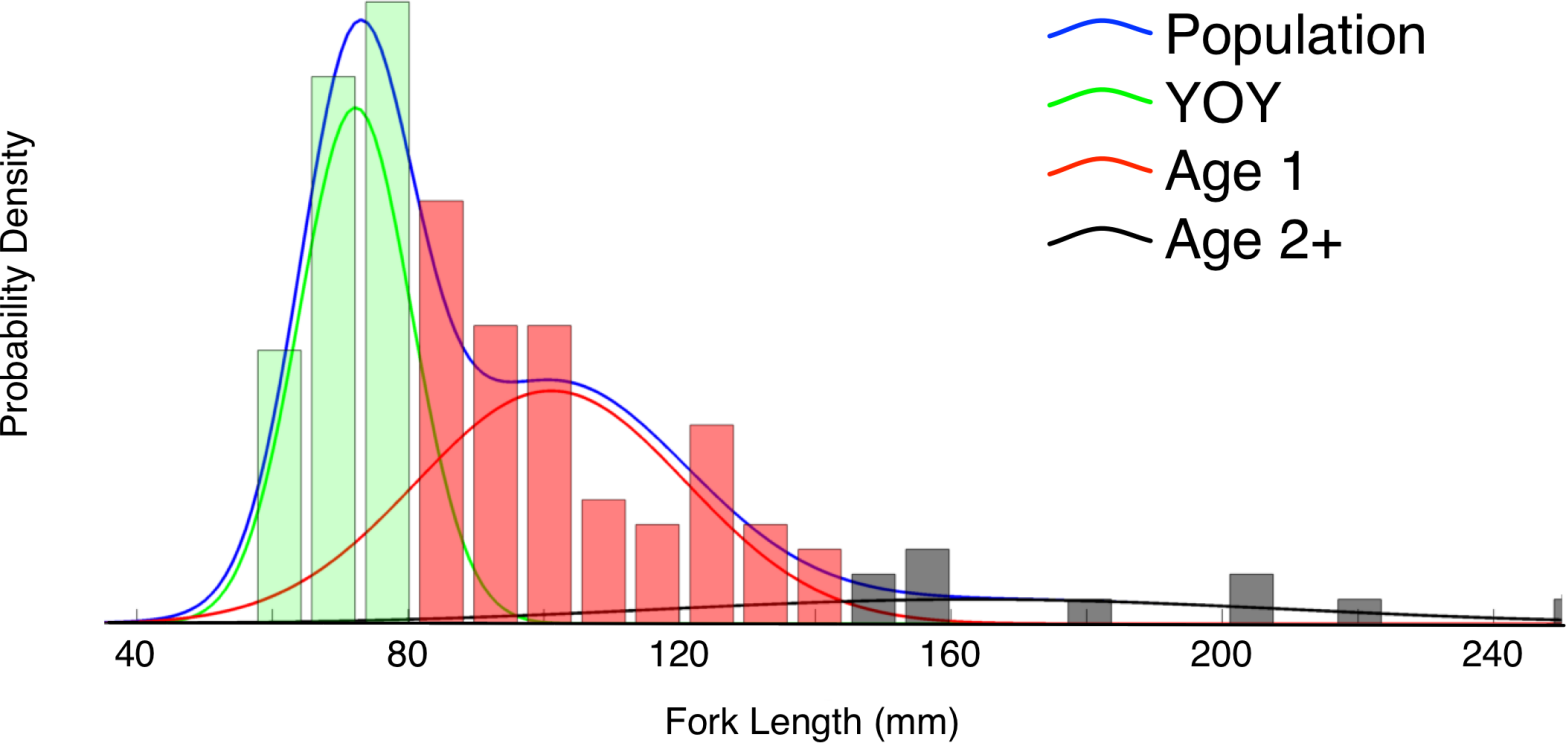
Example of the fitting procedure for estimating age and length distributions from October survey data. Blue curve is the probability density of the Gaussian mixture distribution representing the entire sample (bars). Green, red and black curves are the individual components representing size distributions of YOY, age 1 and age 2+ fish, respectively. The shading of the bars represents the component to which each individual was assigned.

We estimated the October abundance of wild juveniles at each site in each year from 3-pass depletion counts as in [36]; a few site-years with only 2 passes were estimated as in [37]. The proportion of various age classes at each site in each year was then estimated from length-frequencies of the captured fish using a disciplined, data-driven approach: We fit a statistical mixture-model in which fish lengths are distributed as a mixture of *K* normal distributions, where *K* is the number of age classes. The mixture distribution for length $L_{t,j}^{i}$ of individual *i* in year *t*, site *j*, was

$$L_{t,j}^{i}∼\sum_{k=0}^{k=K}\alpha_{k}N(\mu_{k}{,\sigma }_{k})$$ (1)

where *k* indicates age class and *α*_*k*_*N*(*μ*_*k*_,*σ*_*k*_) is a weighted normal distribution of fish lengths with estimated mean *μ*_*k*_, sd *σ*_*k*_ and relative abundance *α*_*k*_ [38]. We treated the number of age-classes *K* as another estimated parameter subject to model selection, by fitting models with one to four age classes to each site-year and selecting the model with the lowest BIC (Bayesian Information Criterion) (Fig 3). We used BIC rather than AICc because we considered our candidate models to include the true model of size and age rather than just the best-approximating model. Lengths and ages of uncaptured (*i*.*e*. unobserved) fish were assumed to be from the same distribution (Eq 1) as observed fish.

By separately parameterizing 9 sites per year, we captured spatial heterogeneity in growing conditions and size-at-age. Annual abundance was estimated as the mean annual density (fish per longitudinal meter of channel) of age 0 or young-of-the-year fish (hereafter YOYs) across the 9 sites, multiplied by the length of channel between the upper dam and the estuary. Sizes and YOY abundances of captively-reared juveniles were estimated similarly, using lengths from a subsample of fish measured at the time of release (usually February or March depending on river conditions).

### Estimating YOY size-distribution on 1 April

Most smolts migrate downstream in April and May; as a simplification we assumed that smolting size = size at ocean entry = YOY size on 1 April of each year. Captively-reared fish were generally released to the river in Feb-Mar. For simplicity we assumed abundance, age- and size-distribution of juveniles at 1 April were the same as at the time of release. This was justified by the faster growth rates observed in the rearing facility than in the wild, suggesting only minor proportional error from ignoring slow growth after release.

Wild fish required a more elaborate treatment since available data were collected the previous October, requiring assumptions about 5+ months of growth and survival. For survival, we simply assumed that mortality between October and 1 April would be absorbed into the corresponding transition rate of the smolting model (see *q*_1_ and *q*_3_, next section). For growth, we adapted a bioenergetics model [39, 40, S1 Appendix] to project growth in a two-step procedure. First, we estimated two parameters (*β* and *η*_*H*_, see below) for each site-year by fitting the model to the period from spring hatching to the time of October surveys. Then, we used the parameters to project growth forward to 1 April at each site.

The initial version of the model estimated growth as a function of daily water temperature and a fish activity level that maximized energy gain [39, 40]. This version could not account for the site-level variability observed in YOY sizes, which commonly arises from individual differences in energy assimilation [19, 40, 41]. We revised the model to assume the variability was generated by size-structured, asymmetric competition, in which a fish’s energy gains depend on its body size relative to other *O*. *mykiss* at the site. Asymmetric energy gains are produced by size-hierarchies in many species, including salmonids [42–44].

In the model, asymmetric competition between a fish of size *L* and a competitor of size *λ* has the form

C(L|λ)=exp⁡(β(λ−L)) (2)

where *β* determines the magnitude of the asymmetry [38]. Larger *β* implies greater competitive dominance of larger fish over smaller fish (Fig A in S2 Appendix). The total competitive cost to each fish was summed over all its competitors at the site, and then rescaled to a competition term in the bioenergetics model, using an additional parameter *η*_*H*_, describing total food availability at the site (see S1 Appendix for details). We estimated *β* and *η*_*H*_ of each site-year for the period from spring “birth” to the October survey as described in the S1 Appendix, and then projected growth forward to 1 April (see Fig B in S2 Appendix for an example).

In using this approach, we effectively assumed that seasonal changes in competition and food conditions were negligible. In a nearby creek, Hayes et al. [45] reported that YOY grow linearly for at least the first 10 months after emergence, consistent with this assumption. However, growth during the spring period of high flows (Feb–Apr) was not well characterized for this age-class, and was observed to support faster seasonal growth for older fish (age 1+), suggesting that our assumption may somewhat under-estimate size on 1 April. If true, the underestimate would artificially weaken our ability to distinguish the predictions of the size-conditional and fixed-rate models described in the next section.

### Models of smolting and marine survival

Scale-analyses in the 1980s suggested a large majority of returning adults smolted at the end of their first year [31]. For simplicity, we assumed juveniles older than YOYs make a negligible contribution to production of adult steelhead. The annual number of wild YOY was estimated as mean YOY density (m^-1^ of channel) at the survey sites, extrapolated to the section of river between the ocean and Los Padres Dam. This estimate includes any translocated fish that survived to October. As noted earlier, the estimate is biased low by perhaps as much as 60%, due to omission of tributaries and the mainstem above Los Padres Dam.

### Fixed-rate model

In the fixed-rate model, we assumed that probability of smolting, surviving, and returning as an adult was constant across years and individuals, and that adult abundance was solely a function of prior juvenile abundance. Management activities such as translocations, which focus solely on improving juvenile survival, are implicitly based on this model of adult abundance. In general, steelhead may spend 1 to 4 years in the ocean before returning to spawn, but analyses of scales in the 1980s suggested that adult steelhead in the Carmel River usually return after 1 or 2 years at sea, in approximately equal proportions [31]. Similar proportions were observed historically in a nearby coastal creek [46], where 53% of adults returned after 1 year and 47% after 2 years. We assumed similar proportions, and predicted annual adult abundance as

$${\bar{A}}_{t}=q_{1}({0.53J}_{t-1}+{0.47J}_{t-2})+q_{2}({0.53C}_{t-1}+{0.47C}_{t-2})$$ (3)

where *J*_*t*_ is the number of wild YOY in October prior to year *t*, and *C*_*t*_ is the number of captively-reared YOY released in year *t* (depending on the precise timing of release they may be age 1 at that point).

The apparent transition rates *q*_1_ and *q*_2_—estimated by least-squares regression—represent confounded products of survival, smolting rate, and the statistical biases in *A*_*t*_ and *J*_*t*_ described in the section on datasets. Although *A*_*t*_ is probably negatively biased, the number of captively-reared fish *C*_*t*_ is unbiased, and so *q*_2_ represents the product of smolting rate, marine survival, and the proportional bias in *A*_*t*_ (Table 2); it should be less than 1.0. The transition *q*_1_ also incorporates overwinter survival and the reciprocal of proportional bias in *J*_*t*_, and may be greater or less than 1.0.

**Table 2:** Interpretation of the apparent transition rates *q*_*x*_.

	Fixed-Rate Model		Size-Conditional Model	
Product of:	*q*_1_	*q*_2_	*q*_3_	*q*_4_
Reciprocal of Bias in *J*_*t*_	Y		Y	
Overwinter Survival	Y		Y	
Smolting Rate	Y	Y	*	*
Marine Survival	Y	Y	*	*
Bias in *A*_*t*_	Y	Y	Y	Y

*J*_*t*_, abundance of wild YOYs in year *t*; *A*_*t*_, counts of adults in year t; Y, Yes absorbed into transition rate

*, *a priori* assumption of the model, but inaccuracy of the assumption is absorbed into the transition rate.

### Size-conditional model

For the size-conditional model, we assumed that smolting probability and early ocean survival were not fixed, but rather functions of individual body size on 1 April. We defined the functions *a priori* from published accounts of other steelhead populations. Smolting probability was modelled as an S-shaped logistic curve with size threshold (inflection point) at *L*_*s*_ = 120 mm and dispersion parameter *σ*_*s*_ = 10 mm, based on [16] and [47]. Size threshold is the length at which smolting probability is 0.5 and dispersion describes the steepness of the curve at that point.

For marine survival we adapted a function derived by [17] from data in [21] and [48] for a nearby creek,

$$min\left\{0.35,\frac{0.84}{1+exp(8.657-0.0369L}\right\}.$$ (4)

Here, marine survival is another logistic curve, truncated at a maximum survival of 0.35. The maximum survival was adjusted downward 20% from the value reported by Satterthwaite et al. [17] (0.433), based on an expectation of maximum survival being somewhat poorer in this watershed relative to smolts from the very pristine watersheds of that analysis [17]. However, the adjustment probably had minor effects on our analysis as it would only affect smolts with *L* > 226 mm, which were rare in our dataset.

We predicted adult abundance similarly to the fixed-rate model,

$${\bar{A}}_{t}=q_{3}(0.53J_{t-1}^{*}+0.47J_{t-2}^{*})+q_{4}(0.53C_{t-1}^{*}+0.47C_{t-2}^{*})$$ (5)

but here the predictors for wild and captively reared production, $J_{t}^{*}$ and $C_{t}^{*}$, incorporate the size-conditional survival and smolting rates just described. Thus *q*_3_ and *q*_4_—again estimated by least-squares—are “apparent” transition rates accounting for bias in *A*_*t*_ and *J*_*t*_ as well as overwinter survival, and any misspecification of the projected smolting rate and marine survival (Table 2).

### Analysis of translocated wild fish

To test how fish translocations were related to YOY lengths and abundances the following October, we fit two sets of statistical models and ranked their performance using AICc. The first set used linear regression to predict mean YOY length. Predictors were various combinations of: 1) number of adult spawners the previous spring; 2) number of fish translocations the previous summer; and 3) streamflow at the end of summer.

The second set of models used non-linear regression to predict YOY abundance. Comparisons were based on three competing concepts of density-regulation (Table 3). In the fixed-production model, we assumed YOYs are highly density-regulated and always saturate the available habitat to a fixed capacity *K*, whereas for the proportional model we assumed YOY abundance is density-independent and thus proportional to the number of spawning adults the previous spring. The logistic-capacity model is intermediate, with the proportional effect of spawning adults tapering off when YOY abundance is near *K*. We also examined additional versions in which YOY abundance is augmented by an estimated fraction *s* of translocated fish that survive until October (Table 3, bottom). As with the life-history models, here the estimated parameters are the confounded product of a quantity of interest (*e*.*g*. capacity, per-capita YOY production, survival) and proportional bias in *A*_*t*_ and *J*_*t*_.

**Table 3:** Competing statistical models for YOY Abundance in October.

Model Name	Equation
Fixed Production	${\bar{J}}_{t}=K$
Proportional	${\bar{J}}_{t}={rA}_{t}$
Logistic Capacity	${\bar{J}}_{t}={rA}_{t}\left(1-\frac{{rA}_{t}}{K}\right)$
Fixed Production + Translocations	${\bar{J}}_{t}=K+{sR}_{t}$
Proportional + Translocations	${\bar{J}}_{t}={rA}_{t}+{sR}_{t}$
Logistic Capacity + Translocations	${\bar{J}}_{t}={rA}_{t}\left(1-\frac{{rA}_{t}}{K}\right)+{sR}_{t}$

**Predictor:** ${\bar{J}}_{t}$, Expected number of YOY juveniles in October of year *t*. **Estimated parameters:** *K*, Capacity of river system for wild October YOY; *r*, Production of wild October YOY per spawning adult the previous spring; *s*, Survival of translocated juveniles from release to October. **Independent Variables:** *A*_*t*_, Number of adult steelhead counted in spring of year *t*; *R*_*t*_, Number of juveniles translocated in year *t*.

## Results

### Trends in juvenile fish

In the mixture model, the model-selection procedure for *K* classified 77% of wild juveniles as YOY across all site-years (sd = 11% among site-years). Neither YOY nor older fish showed a significant trend in abundance over time, but did show substantial year-to-year variability—annual YOY density had a coefficient of variation of 46%. Spatial variability was lower (coefficient of variation = 28%). The mean density across all sites in all years was 1.96 YOY per meter of river channel.

**Fig 4:**
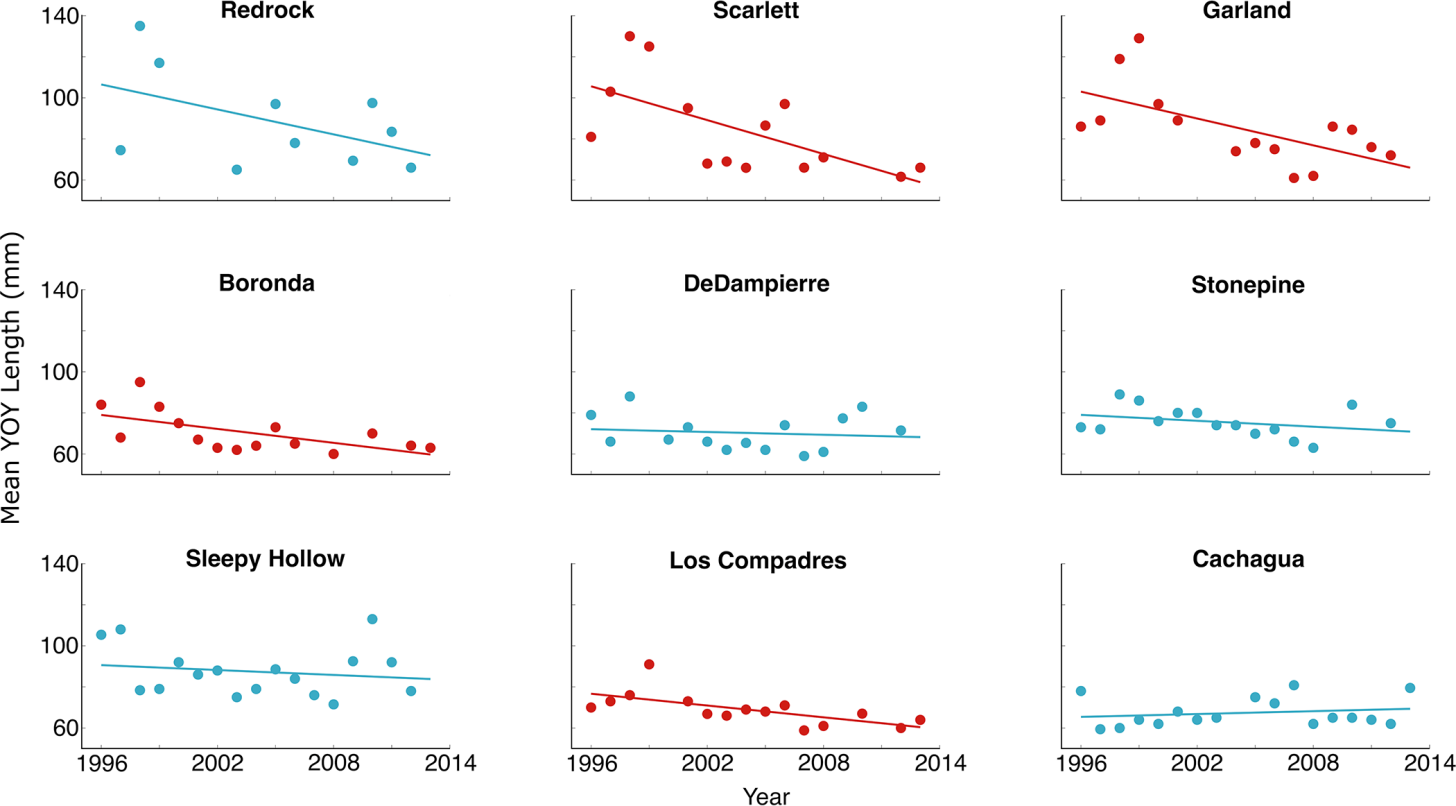
Mean lengths of YOY *O*. *mykiss* at October survey sites, 1996–2013. Sites are ordered horizontally from further downstream (Redrock) to furthest upstream (Cachagua). Significant regression slopes are in red; names of sites as in Fig 2.

In contrast to abundance, the mean length of YOY fish did decline in the river, by an average of 0.98 mm per year since 1996 across all sites. The decline was heterogeneous across the nine survey sites: four showed a statistically significant decline that averaged 2.34 mm per year (Fig 4), one (Redrock) showed a non-significant decline of similar magnitude to these four, and the remaining four were non-significant.

**Fig 5:**
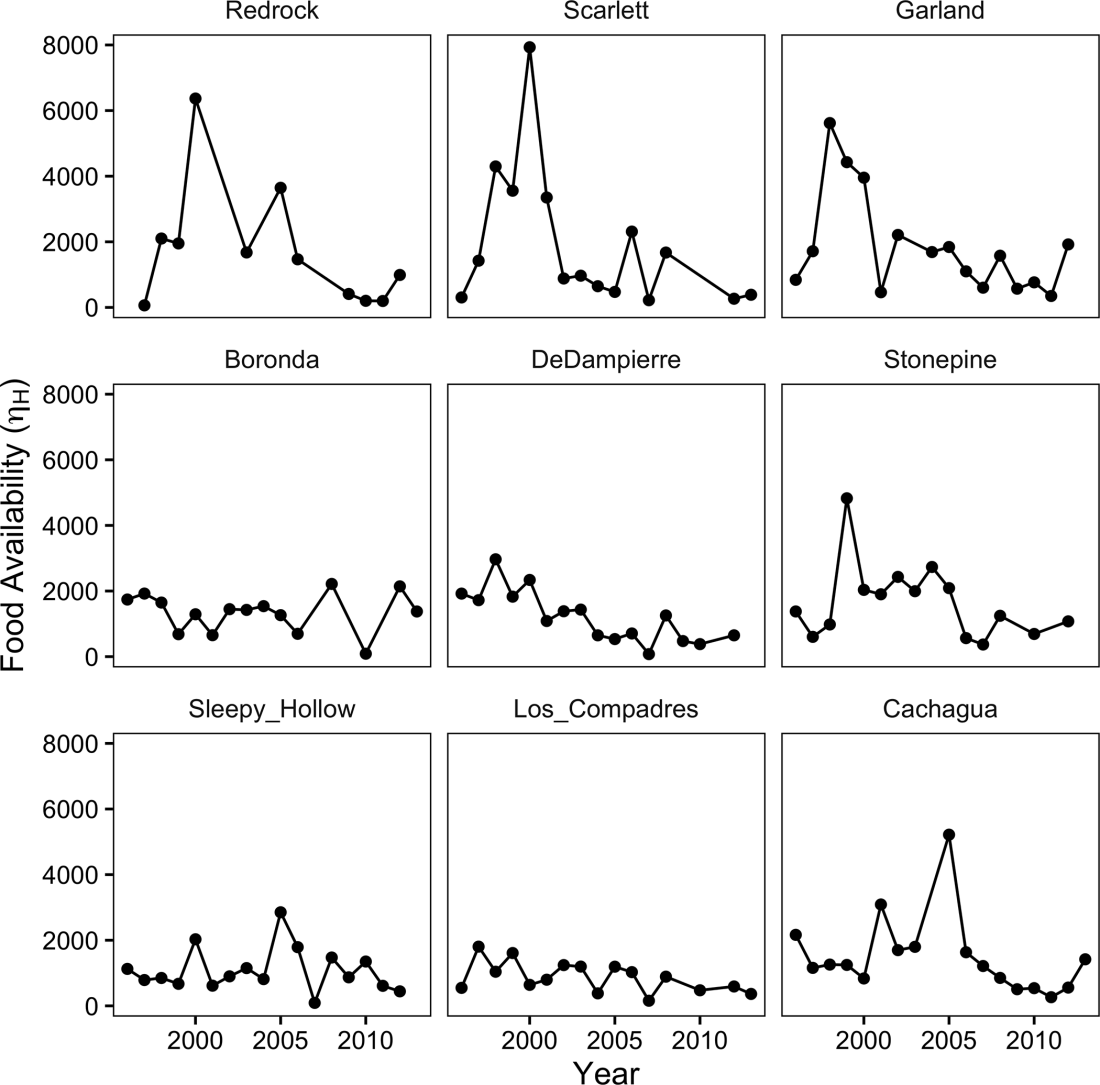
Inferred food availability at nine survey sites during 1996–2013. Sites are arranged horizontally in order from lower to upper river. The site- and year-specific food availability ***η*_*H*_** was an estimated parameter of the bioenergetics model, which was fit to fish-size data and water-temperature data (S1 Appendix).

According to inference from the bioenergetic model, at least some of the decline in growth rates was associated with changes in food availability. The fitted parameter *η*_*H*_, interpreted as site- and year-specific food availability, exhibited an initial pulse followed by decline at several sites in the lower river (top row of Fig 5).

### Comparison of life-cycle models

**Fig 6:**
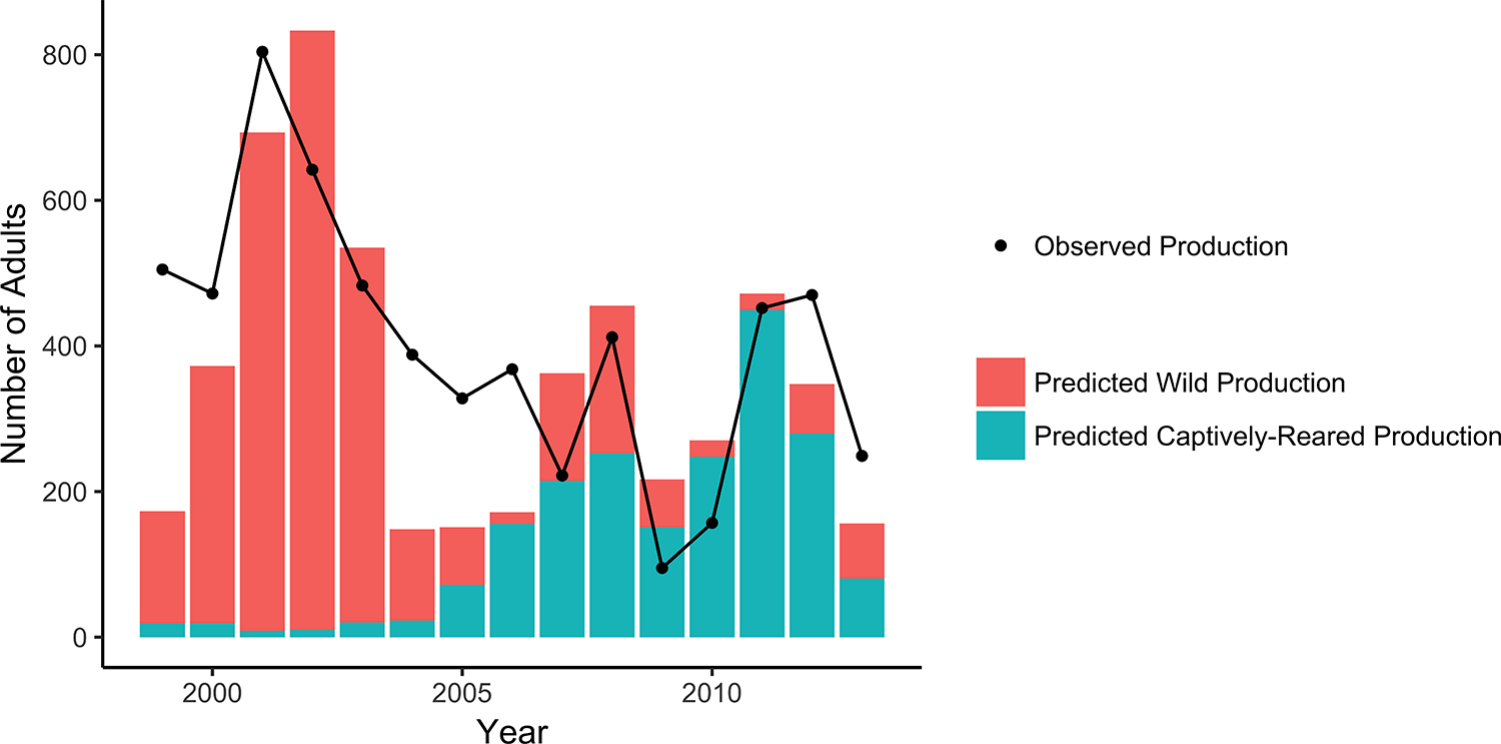
Adult abundances predicted by the size-conditional model. Shown are predictions with apparent transition rates *q*_3_ and *q*_4_.

The size-conditional model outperformed the fixed-rate model, with AICc scores of 202.53 and 205.41, respectively (Table 4). The evidence ratio was 4.2, suggesting that the size-conditional model was over four times as likely as the fixed-rate model to be the best approximating model. The size-conditional model explained 85% of the variance in adult abundance (adj. *R*^2^ = 0.852; see Fig 6).

**Table 4:** Comparison of predictive models for adult abundance.

Model	K	AICc	Δ_*i*_	Wt	Adj. *R*^2^	Evidence Ratio Against
Size-Conditional Model	3	202.53	0.00	0.81	0.852	-
Fixed-Rate Model	3	205.41	2.88	0.19	0.821	4.2

K, number of parameters in model; AICc, Akaike’s Information Criterion, corrected for small samples; Δ_*i*_, difference from AICc of best model; Wt, Akaike weight.

In the top-ranked model, the apparent transition rate for captively-reared production was about 40% less than 1.0 (${\hat{q}}_{4}$ = 0.594, SE = 0.143, *p*[*q*_4_ = 1] = 0.013). This transition rate represented the proportional bias in *A*_*t*_ combined with any inaccuracies in the size-conditional functions of the model (Table 2), and its departure from 1.0 was very close to the expected value of -0.448 based on the spatial-evenness assumption described in the methods. This finding in turn suggests that inaccuracies in the size-conditional functions were rather modest.

The apparent transition for wild production *q*_3_ reflected the above biases as well as the bias in *J*_*t*_ and the effect of overwinter survival (Table 2). If overwinter mortality were negligible and the spatial-evenness assumption explained all proportional bias (*i*.*e*. were true for both spawning and for October distribution of “future” smolts), *q*_3_ would be approximately

$$\frac{1-0.448}{1-0.615}=1.43$$

In fact its estimate was about 18% less (${\hat{q}}_{3}$ = 1.178, SE = 0.158), reflecting both overwinter mortality and our finding that the distribution of fast-growing “future smolts” can be spatially heterogeneous (Fig 4).

In the second-ranked model (fixed-rate), adult abundance was positively associated with abundance of wild juveniles (${\hat{q}}_{1}$ = 0.0076, SE = 0.0012, *p <* 0.0001), but negatively associated with abundance of captives (${\hat{q}}_{2}$ = -0.0433, SE = 0.0147, *p* = 0.0115). Although second-ranked, this fixed-rate model still enjoyed modest support (Δ_*i*_ = 2.88, weight = 0.19). Contrary to the top-ranked model, it suggested negative impacts from captive rearing. Indeed the per-capita magnitude of this negative relationship is about 5.7 times greater (= 0.0433÷0.0076) than the positive relationship with wild juveniles.

### Contribution of captively-reared fish

**Fig 7:**
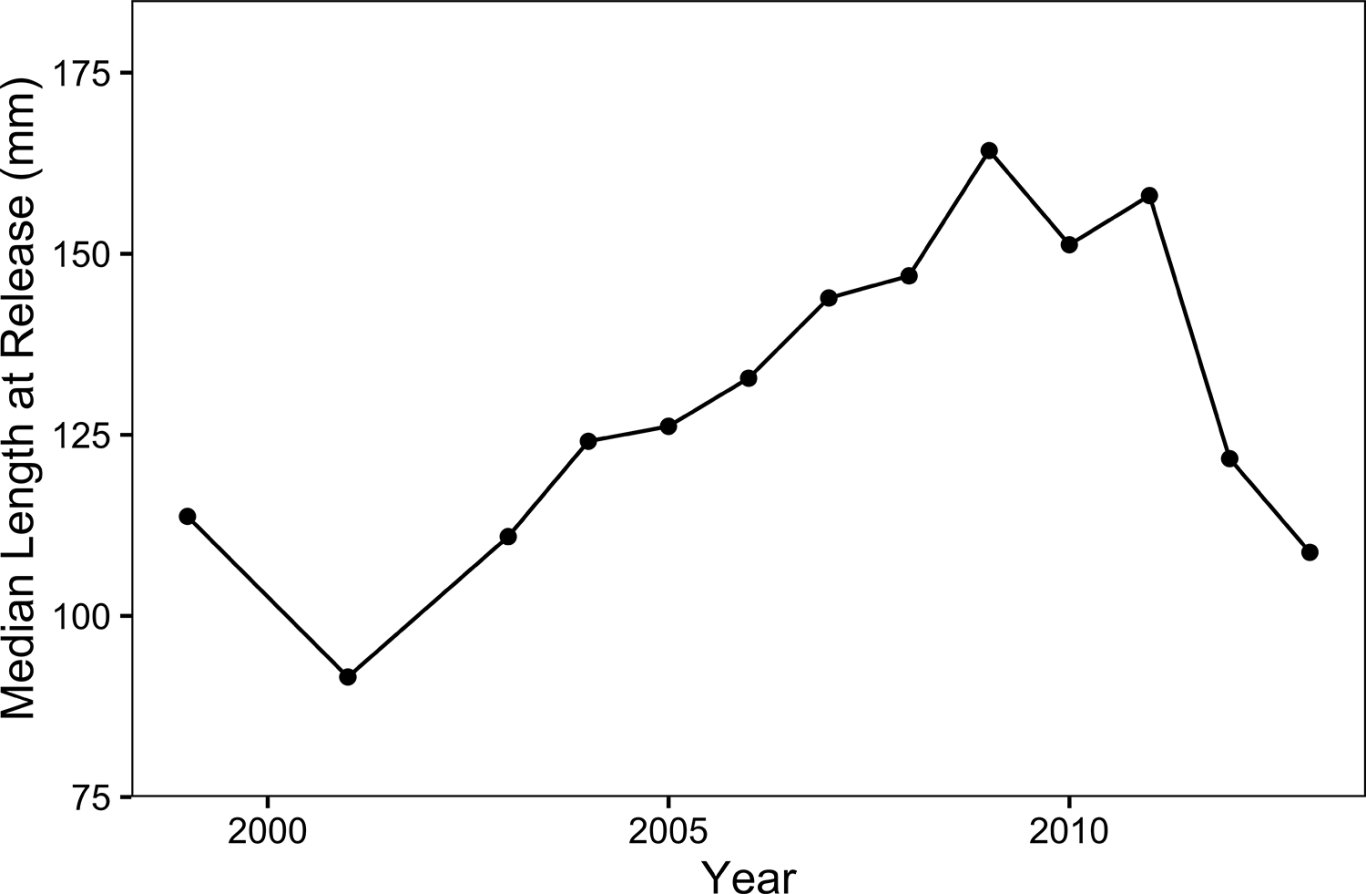
Median lengths of steelhead at the time of release from captive-rearing. A change in feeding practices at the rearing facility, emphasizing abundant krill to reduce cannibalism among *O*. *mykiss*, substantially increased the size of released fish over the course of the study. Declines in release size after 2011 were probably due to poorer rearing conditions in the facility and early releases, both occasioned by drought effects on operations.

According to the size-conditional model, the composition of migrant adult steelhead shifted dramatically after 2005 (Fig 6). Before 2005, the vast majority of adults reared in the river as juveniles, but after 2005 most came from the rearing facility. This shift was driven partly by the declining growth rates of wild juveniles described earlier (Fig 4), and partly by an increase in growth rates of captively-reared fish, caused by a change in operations at the rearing facility (Fig 7). The size-conditional models suggested that without this change in operations, the steelhead run in Carmel River would be very small indeed (*i*.*e*. small blue bars throughout Fig 6). Thus, the top-ranked model inferred a very different role for captive-rearing (supporting anadromy) than the second-ranked model (negatively associated with anadromy, ${\hat{q}}_{2}$ = -0.0433).

### Translocated juveniles and YOY length

The relationship between translocations and subsequent YOY body length was somewhat ambiguous. The top-ranked model as judged by AICc had only streamflow as a predictor (Table 5), but the second-ranked model was nearly as good (Evidence ratio against = 1.3; Table 5), and included two predictors: the number of adult spawners the previous spring and the number of translocated juveniles the previous summer. In the top-ranked model, an increase of streamflow by 1 ft^3^s^-1^ was associated with an increase of fish length by 1.15 mm (Table 6, top), but only explained about a third of the total variance in length (adj. *R*^2^ = 0.35). In the second-ranked model (Table 6, bottom), the number of adults was actually *positively* associated with YOY length, opposite of what would be expected if larger numbers of spawners induced higher YOY densities, greater competition, and slower growth. The number of translocated fish, on the other hand, was negatively associated with YOY length (Table 6, bottom), which was consistent with the competition hypothesis. The magnitude of the effect was a loss of 0.6 mm of length per 1000 translocated fish. This model explained slightly more of the length variance (adj. *R*^2^ = 0.41) than the flow model.

**Table 5:** Models for YOY Length in October, ranked by AICc.

Model	K	AICc	Δ_*i*_	Wt	Adj. *R*^2^	Evidence Ratio Against
Flow	3	129.53	0.00	0.35	0.353	-
Adults + Translocations	4	130.02	0.49	0.27	0.412	1.3
Adults + Flow	4	131.03	1.50	0.16	0.378	2.2
Translocations + Flow	4	131.36	1.82	0.14	0.367	2.5
Translocations	3	133.63	4.10	0.04	0.188	7.8
Fixed (Intercept only)	2	135.54	6.01	0.02	N/A	20
Adults	3	136.06	6.52	0.01	0.070	26

K, number of parameters in model; AICc, Akaike’s Information Criterion, corrected for small samples; Δ_*i*_, difference from AICc of best model; Wt, Akaike weight.

**Table 6:** Coefficients for best models of YOY length in October.

Model	Term	Estimate	Std. Error	*p*
Flow^a^	Intercept	67.82	3.42	<0.0001
	Flow	1.15	0.36	0.006
Adults + Translocations^b^	Intercept	72.50	4.14	<0.0001
	Adults	0.023	0.0085	0.018
	Translocations	-0.0006	0.0002	0.006

Flow, streamflow in ft^3^s^-1^ on 30 September at USGS Gauge 11143200; Adults, adults steelhead counted at San Clemente Dam the previous spring; Translocations, total number juveniles translocated the previous summer.

^a^ Adjusted *R*^2^ = 0.35; *p* = 0.006.

^b^ Adjusted *R*^2^ = 0.41; *p* = 0.007.

The third- and fourth-ranked models also had substantial support (Δ_*i*_ < 2), and simply involved other combinations of the same three predictors—flow, translocations, and number of adults. A model with no predictors (intercept only) had an evidence ratio of 20 against (Table 5), indicating considerably less support (Δ_*i*_ > 4). Thus, there is a detectable yet unresolvable (and modest) relationship between all three predictors and the October size of juveniles.

### Translocated juveniles and YOY abundance

For juvenile abundance in October, the top-ranked model was logistic-capacity + translocations (Table 7). It was about three times more likely to be the best model than the second-ranked model, which was fixed-production (evidence ratio of 3.2; Table 7). The estimated terms for the logistic curve showed that when number of spawners was low, about 270 “apparent” wild juveniles would be expected in October for each adult spawner the previous spring (Table 8, term *r*). Correcting for the biases in *J*_*t*_ and *A*_*t*_ via the spatial-evenness assumption, true *r* would be closer to 193 (= 272.5÷1.41). When the number of spawners was high the number of apparent wild juveniles was predicted to level off at around 283,000 (Table 8, term *K*). The true capacity would actually be as high as 734,000 if proportional bias in *J*_*t*_ approaches -0.615 (evenness assumption).

**Table 7:** Models for YOY abundance in October, ranked by AICc.

Model	K	AICc	Δ_*i*_	Wt	LL	Evidence Ratio Against
logistic capacity + translocations	4	422.77	0.00	0.61	-205.84	-
fixed production	2	425.09	2.32	0.19	-213.54	3.2
fixed production + translocations	3	425.48	2.71	0.16	-208.88	3.9
logistic capacity	3	428.23	5.46	0.04	-210.26	15
proportional + translocations	3	433.60	10.83	0.00	-212.94	225
proportional	2	438.43	15.66	0.00	-216.81	2500

K, number of parameters in model; AICc, Akaike’s Information Criterion corrected for small samples; Δ_*i*_, difference from AICc of best model; Wt, model weight; LL, log-likelihood.

**Table 8:** Coefficients for best model of YOY abundance in October.

Model	Term	Estimate	Std Error	*p*
logistic capacity + translocations	*s*	1.90	0.62	0.0076
	*r*	272.5	44.6	<0.0001
	*K*	282,676	39,538	<0.0001

*s*, survival of translocated juveniles; *r*, wild juveniles per adult; *K*, capacity of river for juveniles.

In the top-ranked model, translocations were positively associated with juvenile abundance the following autumn (Table 8, term *s*), suggesting that translocations were having the desired effect of raising juvenile abundance. The term *s* can be interpreted as the proportion of translocations surviving until the October survey (Table 3, bottom half), but also incorporated the bias in *J*_*t*_. The apparent magnitude of the abundance response was much greater than 1 (1.9 October fish per translocated fish, term *s* in Table 8). However, if *J*_*t*_ has a -0.615 bias as suggested earlier, the true value would be closer to 0.73 October fish per translocated fish, suggesting substantial survival of translocated fish.

The second- and third-ranked models also enjoyed some modest support, with evidence ratios of 3.2 and 3.9 against, respectively (Table 7). Both were fixed-production models, further supporting the idea of strong density-dependence during the first year of life—in this case so strong that even low numbers of adult spawners apparently saturated the available habitat for juveniles. Along with the fourth-ranked model (logistic capacity), the summed weights for models with apparent density regulation was > 0.99 (Table 7), indicating strong support for the phenomenon. The summed weights for models with detectable effects of translocations was 0.77, somewhat weaker support but still suggesting odds of 3.3 [= 0.77÷(1–0.77)] that translocations successfully raised the overall abundance of juveniles.

## Discussion

The results illustrated both the rewards and the discontents of analyzing population responses to conservation actions. The data often clearly favored a particular model without quite excluding its competitors from consideration. Within our framework of AICc, model selection and evidence ratios, we often found evidence ratios of 3 to 4 against a lesser-ranked model. This corresponds to a non-negligible probability of 0.2–0.25 that the lower-ranked model was in fact the better predictor for future population responses.

The size-conditional model gave remarkably good predictions of adult abundance, especially considering the specificity of the *a priori* assumptions about size-dependence, the biases in the data, and the omission of any variability in marine conditions. However, the fixed-rate model also enjoyed modest support and made nearly as good predictions. Unfortunately, the implications of the second-ranked model (negative effect of captive rearing) are diametrically opposed to those of the top-ranked model (a positive contribution from captive rearing). It is possible the release of large captively-reared smolts into the lower river may be having an outsized negative impact on production or survival of wild smolts. The mechanism would have to be intense, operating only in the lower river, estuary or nearshore during the one or two months between release and migration season. However, the negative association could very well be spurious, because the annual number of captives appears to be negatively associated with streamflow (*r* = -0.46, *p* = 0.056), which itself is correlated with larger wild YOYs (*r* = 0.62, *p* = 0.006; also Table 5) and by implication, smolt production and survival. Indeed, it was the intent of the captive-rearing program to counteract the effects of low streamflow and its effects on the population, so some of the weight for this model can surely be interpreted as a prior probability for the spurious correlation. Further clarity on the effect of captive rearing would likely require tagging studies that allow return rates of individual smolts to be directly observed.

According to the top-ranked model, the decline of adult Carmel River steelhead appears to be driven by a decline in freshwater growth rates of wild-reared fish, such that adult steelhead runs have come to be dominated by captively-reared fish. Model comparisons provide strong evidence that abundance of wild juveniles is density-regulated, and more modest evidence that wild fish translocations successfully raised the abundance above carrying capacity.

The effects of the translocations on YOY growth are less clear, because they are confounded with the effects of streamflow on growth—greater summer streamflow is associated with faster growth, as is commonly observed in other salmonid populations [49–53]—but here it is also associated with fewer translocations because less of the river channel is dry. The statistical model that omitted flow (Table 6, second model) gave a sense of the maximum effect that translocations might have on fish growth—on the order of YOY smaller by 6 to 12 mm in years with translocations of 10,000 to 20,000 fish. This might be enough to reduce adult abundances slightly, but not enough to explain the declines depicted in Fig 4.

### Why the declines in growth rates?

Why has a concerted effort to improve river conditions over the past 15 years been accompanied by a decline in growth rates of wild YOY steelhead? While our results do not answer this question, they do help us frame some hypotheses.

**Fig 8:**
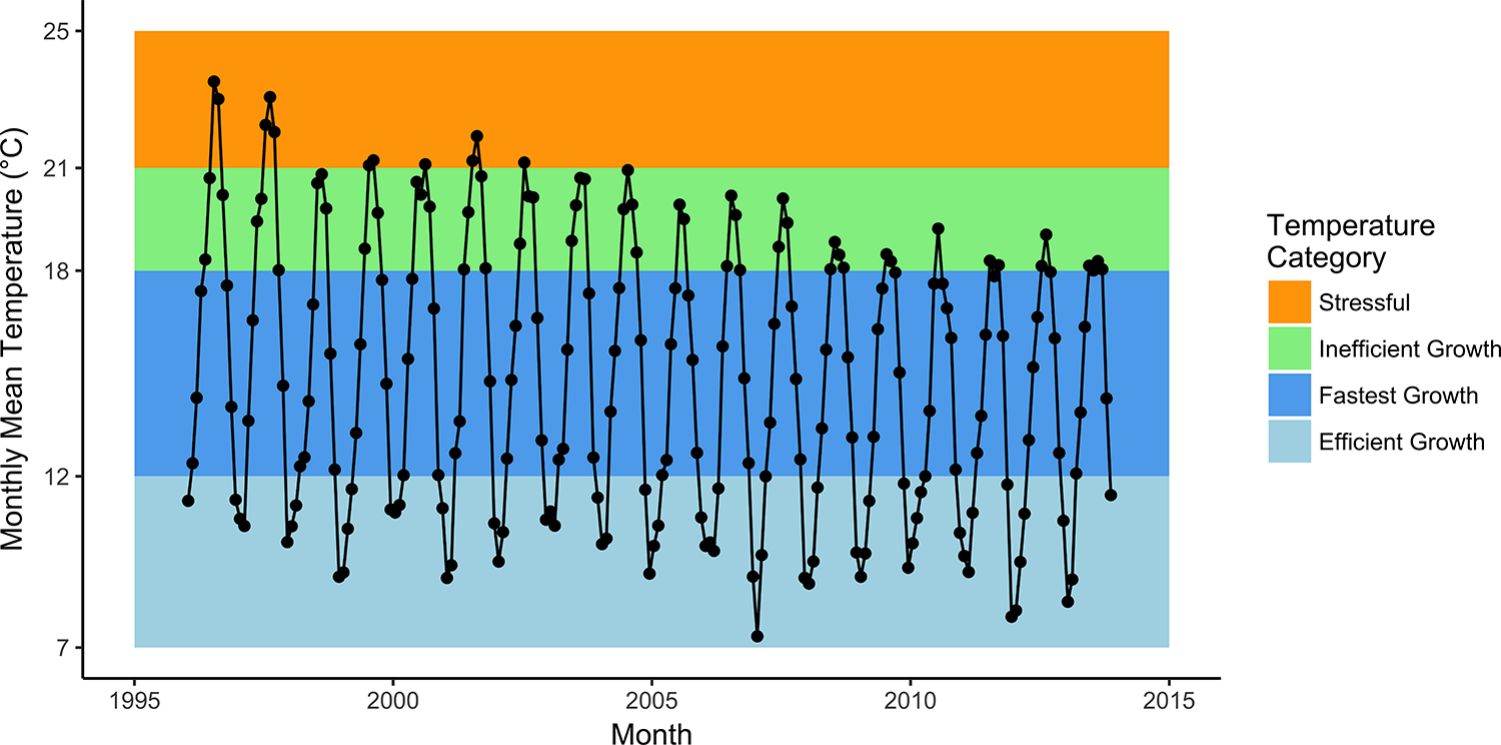
Monthly mean temperature of Carmel River during 1996–2013. Also shown are temperature categories for steelhead rearing habitat, adapted from [40] and the bioenergetics model; see text for further description.

One result of riparian restoration has been a denser riparian tree canopy, accompanied by a decreasing trend in river temperature (Fig 8). The potential effects of the trend on YOY growth are intricate due to amplitude of the annual cycle of temperature (depicted by monthly means in Fig 8) interacting with the bioenergetics of growth for steelhead (colored bands in Fig 8). Growth response to temperature in salmonids is typically hump-shaped, with fastest growth (within 85% of the maximum) approximately in the range of 12° to 18° C. Below that range growth is slower, but relatively efficient, because basal metabolism consumes less energy at lower temperatures. Above that range growth is also slower but inefficient, as increasing proportions of energy reserves are consumed by basal metabolism, which increases exponentially with temperature. Above 21° juveniles begin to retreat to thermal refugia or express heat-shock proteins, indicative of thermal stress [40]. In the Carmel River we find that the trend in temperature has reduced the time each summer that juveniles must spend in stressful conditions (Fig 8, orange band), and increased the opportunity for realizing their fastest (or nearly fastest) growth rates (Fig 8, dark blue and lower part of green bands). Thus, it seems likely that without the riparian restoration and decline in water temperatures the reduction of growth rates may have been even more severe.

Secondly, the bioenergetic model infers at least some role for declining food availability (Fig 5). The greatest growth in the lower river was observed during 1997–1999, on the heels of two exceptionally wet winters in 1995 and 1998, which produced the two largest peak flows in the Carmel River since at least 1962 (USGS gauge 11143250). Perhaps these disturbance events altered habitat or food webs so as to temporarily improve conditions supporting juvenile growth. Stream systems in this region have “episodic” hydrologic regimes [54, 55], characterized by generally low-precipitation conditions punctuated by extremely wet years with a return interval of one or two decades. These high-flow years can rework the channel and regenerate pool habitat for juvenile steelhead that fills in or degrades in intervening years [56]. Perhaps the population response observed in Fig 1, of steep improvement followed by long decline, is completely natural and typical of episodic steelhead streams. It is also possible that the increased shading of the stream has reduced the primary production in the stream, which in turn reduced an invertebrate food source for the fish. However, the increase in shading over time would be gradual, while the decrease in food availability appears to be rather abrupt.

Another pivotal event occurred in 2000 when Federal regulations phased-out most organophosphates for use as residential insecticides. This has led to a subsequent increase in the use of pyrethroid insecticides in many agricultural and suburban landscapes in California [57, 58]. Unlike organophosphates, pyrethroids are strongly hydrophobic and bind to stream sediments, where they sometimes persist long enough to reach toxic levels for benthic macroinvertebrates [57, 59]. Simulation studies suggest that insecticides with only minor direct effects on salmonids can still have strong indirect effects on growth by affecting availability of invertebrate prey [60]. Ironically, pyrethroids have stronger toxic effects on invertebrates at lower water temperatures [59], so in this scenario the cooler summer water temperatures in the Carmel River, probably caused by recovering riparian vegetation, could actually magnify the toxicity of any pyrethroids present.

Finally, the higher growth rates all occurred prior to an estuary rehabilitation project completed in 2004. Although a major goal of the rehabilitation was to improve and expand estuarine habitats for juvenile steelhead, observations suggest that some of the new habitat has been colonized by anadromous striped bass (*Morone saxatilis*). This introduced species is a major predator of juvenile salmonids in other river systems of the state [61]. Although present in the Carmel estuary since at least the 1980s [31], the large numbers of adults in the rehabilitated estuary habitat and occasional observations of the species in the lower river are recent. It is possible that foraging by steelhead is being suppressed by increased activity and dispersion of a major predator.

## Conclusions

We did not definitively identify a cause for the recent population decline of Carmel River steelhead, but we did find some hypotheses to be more likely than others. The data indicated reduced juvenile growth rates in the lower river, and the model best-supported by the data attributed the population decline to these reduced growth rates and their assumed effect on smolting and survival. If true, then the model implies the steelhead run is now mostly composed of captively-reared fish. A competing model with less support, but still plausible, suggests paradoxically that captively-reared fish have had a strong negative effect contributing to the decline, but we have reason to believe this arises from a spurious correlation; years with higher summer flows apparently require fewer fish to be captively reared, but also may generate faster juvenile growth rates in the river. This is observed in other salmonid populations, and would tend to improve the steelhead run according to the best-supported model. The likely spurious correlation indicates how population management differs from pure research. In a research setting, captive rearing would have been randomized with respect to stream flow to prevent just such a correlation, but this would likely be considered unethical for a threatened species where the goal of captive rearing is to mitigate low stream flows.

Lower Carmel River is an alluvial channel that has been the focus of extensive restoration efforts over the last two decades, so why have juvenile growth rates declined there? Within-river translocations of juveniles from drying reaches probably raised the juvenile abundance above carrying capacity, but appeared to have either negligible or only minor effects on juvenile growth rates, and hence little relationship with adult production. During the study period, the river displayed a downward trend in river temperature, an intended outcome of riparian restoration. Our bioenergetics analysis suggests that if anything this has improved the conditions for growth in the river, shifting the most likely cause to a sharp decrease in food availability. The timing of the sharp decrease suggests a number of hypotheses including: a natural cycle of food availability associated with an episodic flow regime; a regulatory change in permitted household insecticides that might have impacted stream invertebrates; and colonization of restored habitat by predatory striped bass, which may suppress foraging activity by juvenile steelhead. Our results illustrate both the value and the probabilistic nature of retrospectively analysing how populations respond to conservation actions.

## Supporting information

S1 Appendix Bioenergetics model. Description of the bioenergetics model used to project growth of juvenile steelhead from October to 1 April.
(DOCX)

S2 Appendix Additional Figures. (DOCX)

S3 Appendix Datasets. (XLSX)
