# Supplementary material for: Size-conditional smolting and the response of Carmel River steelhead to two decades of conservation efforts
Source: PLoS One. 2017 Nov 30;12(11):e0188971. doi: 10.1371/journal.pone.0188971 (PMC5708832; doi:10.1371/journal.pone.0188971)
Supplement: S1 Appendix — Description of the bioenergetics model used to project growth of juvenile steelhead from October to 1 April. (DOCX) [file pone.0188971.s001.docx]

# S1 Appendix. Bioenergetics Model

The bioenergetic model is adapted from [1, 2], differing only in the addition of a competition term. The model assumes changes in fish weight *W_i_* arose from the difference between food assimilation (first term) and metabolic costs (second term):

$\frac{dW_{i}}{dt}=\Phi_{c}\left( T \right)A\left( t,L \right)c_{1}W_{i}^{c_{2}}-\Phi_{m}\left( T \right)m_{1}W_{i}^{m_{2}}$ (1)

Energetic costs involved mass-specific costs as a function of temperature $\Phi_{m}\left( T \right)$; and an allometric relationship scaling it to total costs as a function of weight, $m_{1}W_{i}^{m_{2}}$. Energy gain (first term) is the product of four quantities: relative energy density of food to fish tissue, *c_1_*; an allometric relationship of consumption and fish weight, $W_{i}^{c_{2}}$; a functional relationship between temperature and maximum consumption, $\Phi_{c}\left( T \right)$; and a competition function $A\left( t,L \right)$ that will be described below. This formulation, except for the competition function, is drawn from [3], [4], and [5] using allometric parameters from steelhead in a nearby stream [6] and temperature scalings from [7] and [8], reparameterized for California steelhead by [4] using data of [9], [10] and [11].

An initial version of Equation (1) (without competition) could not account for the observed variability in YOY fish sizes, which commonly arises from individual differences in energy assimilation [12], [2, 5]. We assumed the variability resulted from size-structured asymmetric competition, in which energy gains of an individual depend on its body size relative to conspecific competitors. Asymmetric energy gains are often produced by size hierarchies in many species, including salmonids [13-15].

Here we assumed that asymmetric competition between an individual of size *L* and a competitor of size *λ* has the form:

*C*(*L*|*λ*) = exp(*β*(*λ* − *L*)) (2)

where *β* determines the magnitude of the asymmetry: Larger *β* implies greater competitive dominance of larger individuals over smaller individuals. The total competitive cost summed over all competitors at the site is thus

$\eta\left( t,L \right)=\int_{L_{b}}^{L_{m}} C\left( L | \lambda\right)n(t,\lambda)\lambda^{2}d\lambda$ (3)

where $n(t,\lambda)$ describes the actual size distribution of all fish at the site at time *t*, $\eta\left( t,L \right)$ is the “effective population density” experienced by an individual of size *L*, and *L_b_* and *L_m_* give the range of fish lengths observed at the site. The $\lambda^{2}$ term accounts for our assumption that competition scales with the fish surface area rather than length. Effective population density is then rescaled to a competition function *A*(*t*, *L*) by assuming that

$A\left( t,L \right)=\frac{\eta_{H}}{\eta_{H}+\eta(t,L)}$ (4)

in which the half-saturation constant $\eta_{H}$ describes the total amount of resources at the site. As $\eta(t,L)$ increases, an individual's access to the resources decreases.

We estimated *β* and $\eta_{H}$ of each site-year for the period from spring “birth” to the October survey, and then projected growth forward to the time of emigration. Estimation required initial and October size distributions and daily water temperatures. October size was from the mixture model; daily water temperatures were linearly interpolated to site locations from four thermal-monitoring sites on the river; and initial size was a truncated normal distribution derived from unpublished data (μ = 34 mm; σ = 5 mm; bounds = 28 mm and 39 mm). After the spawning season, all age 0 individuals were assumed to disperse homogeneously throughout the river [16]. Equation (1) was solved iteratively to identify *β* and $\eta_{H}$ that produced a suitable fit to the initial size distribution, as judged by a Kolmogrov-Smirnov test (K-S test). The identified parameters and additional temperature data were then used to project growth forward to April 1.

**References**

1. Arriaza JL. The use of mathematical models for informing management strategies: An application in steelhead trout and fleet dynamics [Master's report]. Santa Cruz, California: University of California Santa Cruz; 2013.

2. Boughton DA, Harrison LR, Pike AS, Arriaza JL, Mangel M. Thermal potential for steelhead life history expression in a southern California alluvial river. Trans Am Fish Soc. 2015;144(2):258-73. doi: 10.1080/00028487.2014.986338. PubMed PMID: WOS:000352726200003.

3. Hanson PC, Johnson TB, Schindler DE, Kitchell JF. Fish Bioenergetics 3.0 software for Windows. 1997.

4. Railsback SF, Rose KA. Bioenergetics modeling of stream trout growth: Temperature and food consumption effects. Trans Am Fish Soc. 1999;128(2):241-56. doi: 10.1577/1548-8659(1999)128<0241:bmostg>2.0.co;2. PubMed PMID: WOS:000081212900003.

5. Satterthwaite WH, Beakes MP, Collins EM, Swank DR, Merz JE, Titus RG, et al. State-dependent life history models in a changing (and regulated) environment: steelhead in the California Central Valley. Evolutionary Applications. 2010;3(3):221-43. doi: 10.1111/j.1752-4571.2009.00103.x. PubMed PMID: ISI:000276790700001.

6. Satterthwaite WH, Beakes MP, Collins EM, Swank DR, Merz JE, Titus RG, et al. Steelhead life history on California's central coast: Insights from a state-dependent model. Trans Am Fish Soc. 2009;138(3):532-48. doi: 10.1577/t08-164.1. PubMed PMID: ISI:000268501100008.

7. Brett JR. Temperature tolerance in young Pacific salmon, genus *Oncorhynchus*. Journal of the Fisheries Research Board of Canada. 1952;9(6):265-323.

8. Thornton KW, Lessem AS. Temperature algorithm for modifying biological rates. Trans Am Fish Soc. 1978;107(2):284-7. doi: 10.1577/1548-8659(1978)107<284:atafmb>2.0.co;2. PubMed PMID: WOS:A1978FB35900010.

9. Myrick CA, Cech JJ. Temperature effects on juvenile anadromous salmonids in California's central valley: what don't we know? Rev Fish Biol Fish. 2004;14(1):113-23. doi: 10.1007/s11160-004-2739-5. PubMed PMID: ISI:000227515300005.

10. Van Winkle W, Rose KA, Shuter BJ, Jager BI, Holcomb BD. Effects of climatic temperature change on growth, survival, and reproduction of rainbow trout: predictions from a simulation model. Can J Fish Aquat Sci. 1997;54(11):2526-42. PubMed PMID: ISI:000072050700006.

11. From J, Rasmussen G. A growth model, gastric evacuation, and body composition in rainbow trout, *Salmo gairdneri* Richardson, 1836. Dana. 1984;3:61-139.

12. Mangel M, Munch SB. A life-history perspective on short- and long-term consequences of compensatory growth. Am Nat. 2005;166(6):E155-E76. doi: 10.1086/444439. PubMed PMID: WOS:000233576600002.

13. Elliott JM. Shadow competition in wild juvenile sea-trout. J Fish Biol. 2002;61(5):1268-81. doi: 10.1006/jfbi.2002.2154. PubMed PMID: ISI:000180447800015.

14. Metcalfe NB. Intraspecific variation in competitive ability and food-intake in salmonids - consequences for energy budgets and growth-rates. J Fish Biol. 1986;28(5):525-31. doi: 10.1111/j.1095-8649.1986.tb05190.x. PubMed PMID: WOS:A1986C663900001.

15. De Roos AM, Persson L, McCauley E. The influence of size-dependent life-history traits on the structure and dynamics of populations and communities. Ecol Lett. 2003;6(5):473-87. doi: 10.1046/j.1461-0248.2003.00458.x. PubMed PMID: WOS:000182259400014.

16. Snider B, Titus RG. Lower American River emigration survey, October 1996 - September 1997. Stream Evaluation Program Technical Report No 00-2. 2000:iii + 21.
