# Supplementary material for: Size-conditional smolting and the response of Carmel River steelhead to two decades of conservation efforts
Source: PLoS One. 2017 Nov 30;12(11):e0188971. doi: 10.1371/journal.pone.0188971 (PMC5708832; doi:10.1371/journal.pone.0188971)
Supplement: S2 Appendix — (DOCX) [file pone.0188971.s002.docx]

# S2 Appendix. Additional Figures

**
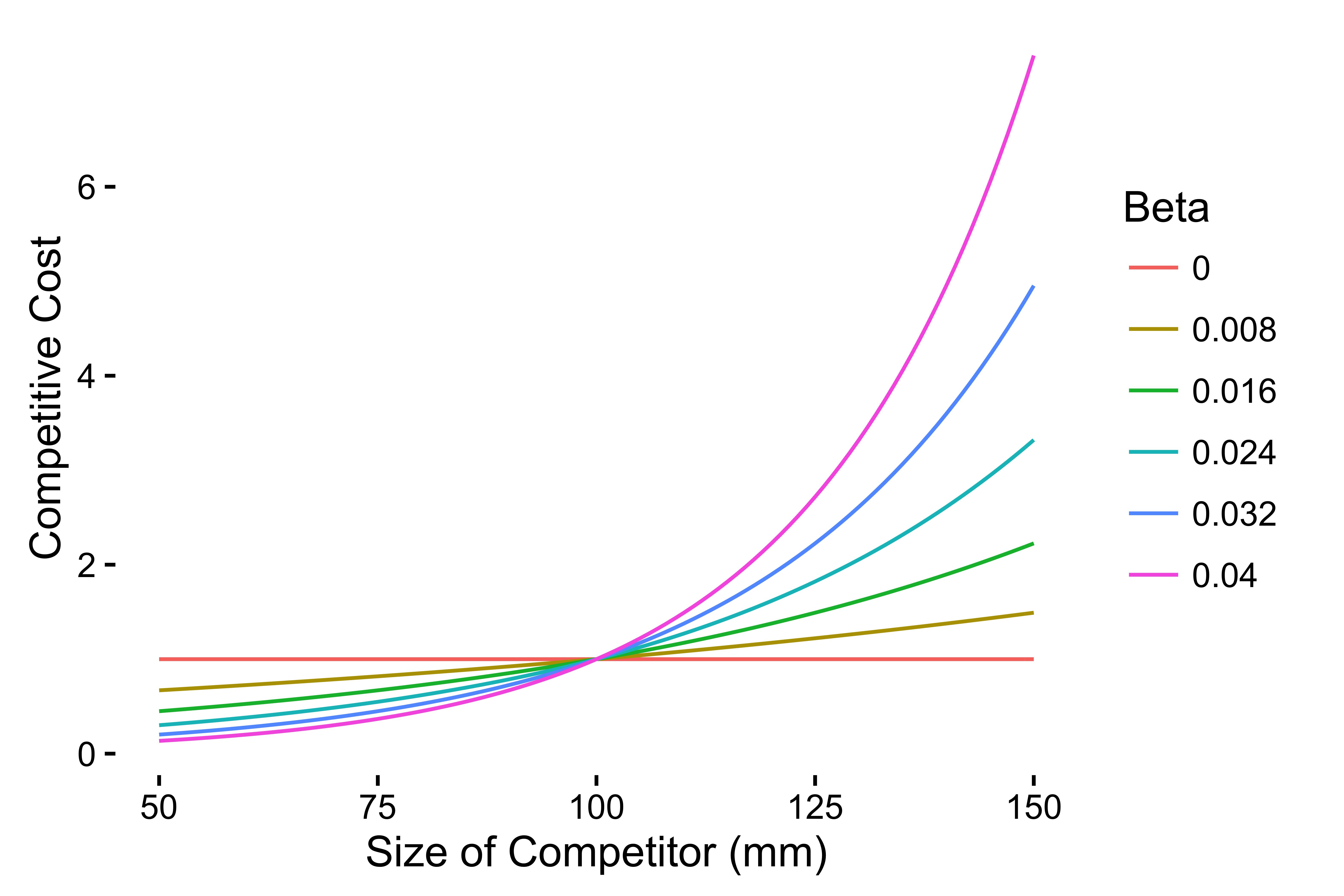
**

**Fig A. Illustration of the competition function for a fish of length *L* = 100 mm.** Competitive cost $C(L|\lambda)$ incurred by the fish as a function of the competitor’s length and the asymmetry parameter $\beta$.

**
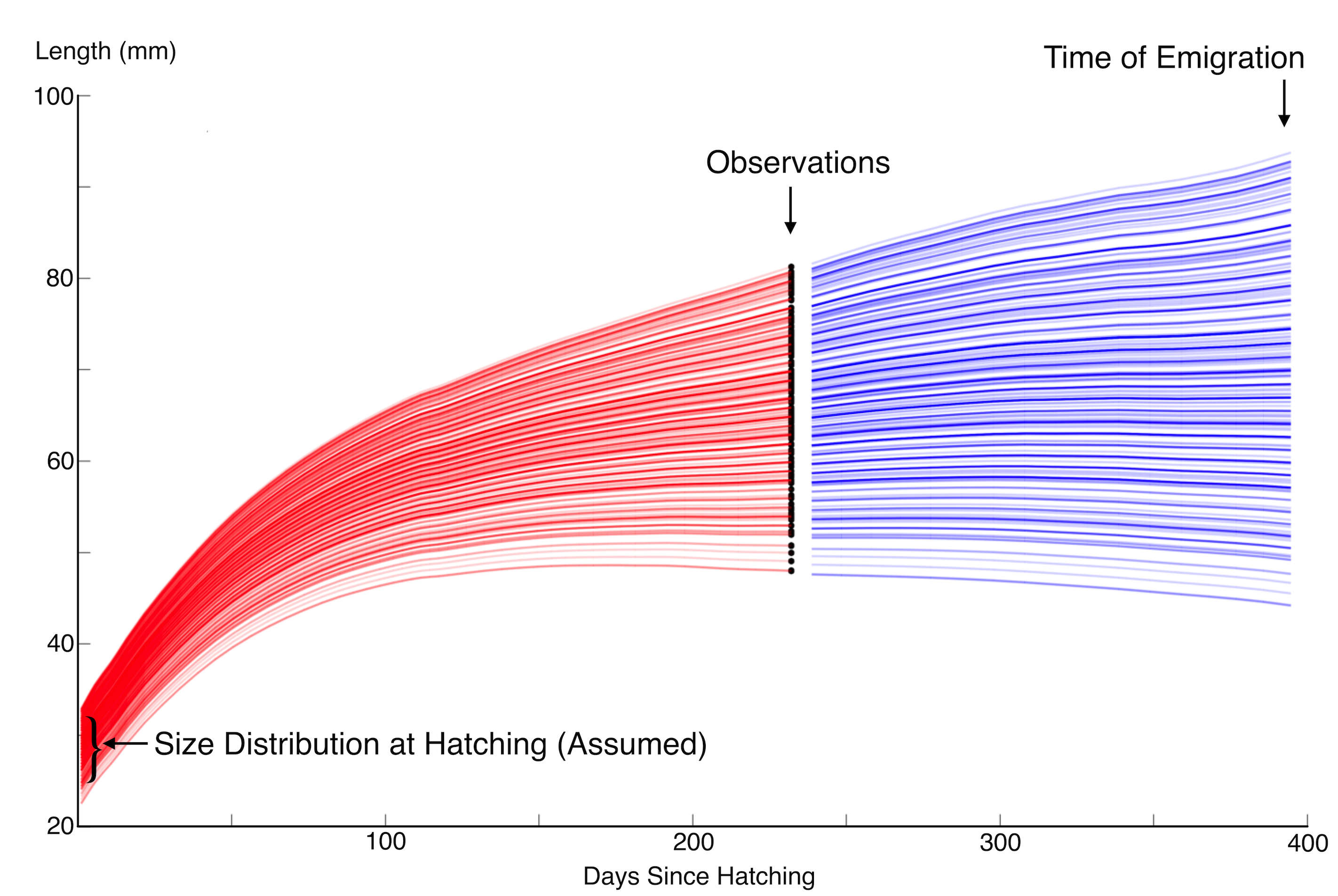
**

**Fig B. Example of the growth-fitting procedure.** The red curves represent reconstructed growth trajectories of individuals observed during the juvenile surveys (black circles); blue curves are the same growth trajectories (same $\beta$ and $\eta_{H}$) projected forward to April 1 (Time of Emigration). Trajectories are based on monitored daily river temperatures and best-fit parameters $\beta$ and $\eta_{H}$ from a K-S test (see S1 Appendix).


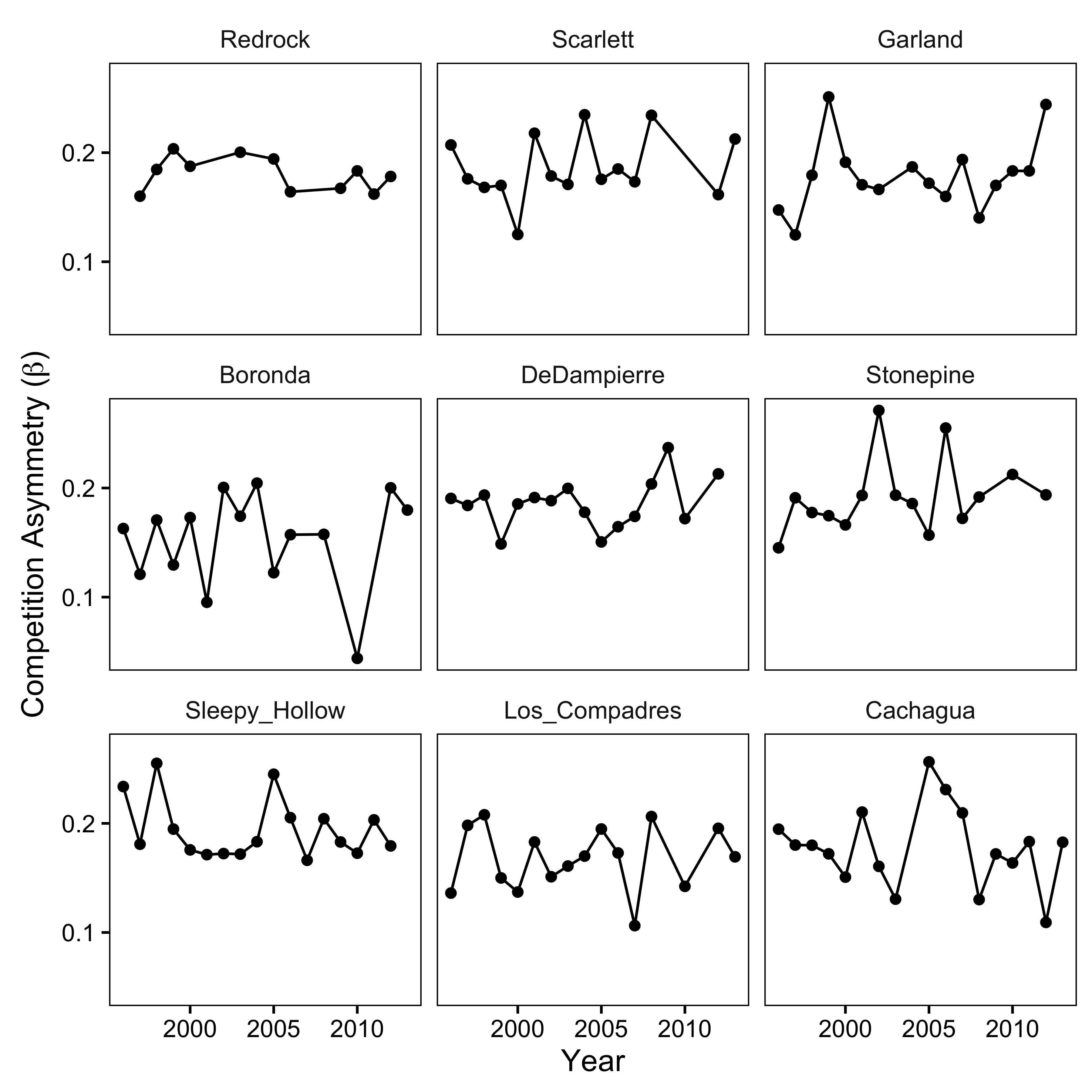


Fig C. Estimated values of the competition parameter *β.* Omitted site-years had no surface flow and zero fish at the time of the October surveys. The completion parameter describes the degree of asymmetry in size-structured competition for food.
